# Approach to the Child and Adolescent With Adrenal Insufficiency

**DOI:** 10.1210/clinem/dgae564

**Published:** 2024-08-18

**Authors:** Giuseppa Patti, Alice Zucconi, Simona Matarese, Caterina Tedesco, Marta Panciroli, Flavia Napoli, Natascia Di Iorgi, Mohamad Maghnie

**Affiliations:** Department of Pediatrics, IRCCS Istituto Giannina Gaslini, Genoa 16100, Italy; Department of Neuroscience, Rehabilitation, Ophthalmology, Genetics, Maternal and Child Health, University of Genova, Genoa 16100, Italy; Department of Pediatrics, IRCCS Istituto Giannina Gaslini, Genoa 16100, Italy; Department of Neuroscience, Rehabilitation, Ophthalmology, Genetics, Maternal and Child Health, University of Genova, Genoa 16100, Italy; Department of Pediatrics, IRCCS Istituto Giannina Gaslini, Genoa 16100, Italy; Department of Neuroscience, Rehabilitation, Ophthalmology, Genetics, Maternal and Child Health, University of Genova, Genoa 16100, Italy; Department of Pediatrics, IRCCS Istituto Giannina Gaslini, Genoa 16100, Italy; Department of Pediatrics, IRCCS Istituto Giannina Gaslini, Genoa 16100, Italy; Department of Neuroscience, Rehabilitation, Ophthalmology, Genetics, Maternal and Child Health, University of Genova, Genoa 16100, Italy; Department of Pediatrics, IRCCS Istituto Giannina Gaslini, Genoa 16100, Italy; Department of Pediatrics, IRCCS Istituto Giannina Gaslini, Genoa 16100, Italy; Department of Neuroscience, Rehabilitation, Ophthalmology, Genetics, Maternal and Child Health, University of Genova, Genoa 16100, Italy; Department of Pediatrics, IRCCS Istituto Giannina Gaslini, Genoa 16100, Italy; Department of Neuroscience, Rehabilitation, Ophthalmology, Genetics, Maternal and Child Health, University of Genova, Genoa 16100, Italy

**Keywords:** adrenal insufficiency, novel treatments, modified release hydrocortisone formulations, optimized hydrocortisone dosing in children, congenital adrenal hyperplasia

## Abstract

The management of adrenal insufficiency (AI) is challenging, and the overall goals of treatment are to prevent life-threatening adrenal crises, to optimize linear growth, to control androgen levels without overdosing in patients with congenital adrenal hyperplasia (CAH), and to improve quality of life in affected individuals. Standard glucocorticoid formulations fail to replicate the circadian rhythm of cortisol and control the adrenal androgen production driven by adrenocorticotropin. To personalize and tailor glucocorticoid therapy and to improve patient outcomes, new pharmacological strategies have been developed that best mimic physiological cortisol secretion. Novel therapeutic approaches in the management of AI include new ways to deliver circadian cortisol replacement as well as various adjunctive therapies to reduce androgen production and/or androgen action/effects. Preclinical studies are exploring the role of restorative cell-based therapies, and a first recombinant adeno-associated virus-based gene therapy is also being developed in humans with CAH. In this article, we present 3 illustrative cases of AI with different underlying etiologies and times of presentation. Diagnostic and management processes are discussed with an emphasis on treatment and outcomes. We have also provided the most up-to-date evidence for the tailored management of children and adolescents with AI.

## Clinical Cases

### Materials and Methods

#### Clinical data

SD Scores (SDS) of height were computed according to Tanner reference charts (1). Body mass index (BMI) SDS was calculated by using the World Health Organization chart (2).

#### Hormonal tests

Hormonal parameters were evaluated by chemiluminescent assay (Roche Elecsys Kit).

#### Brain magnetic resonance imaging

Brain MRI scan was performed on a 3T MRI scanner (Ingenia, Philips) with turbo spin echo, DRIVE, susceptibility-weighted imaging, and fast field echo sequences. Additionally, apparent diffusion coefficient and diffusion-weighted imaging sequences were acquired.

### Case 1

**Figure 1.**
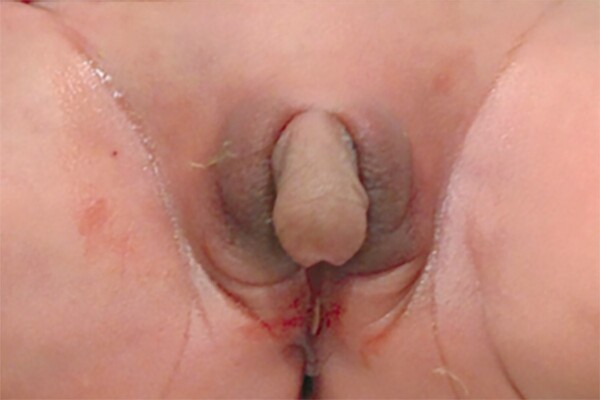
Case report: a 3-week infant with severe clitoral hypertrophy.

A 3-week-old neonate with ambiguous genitalia (Fig. 1) was referred to our department from a local hospital for vomiting and weight loss.

The patient was born by cesarean delivery at 41 weeks of gestational age (GA) from consanguineous parents of Moroccan origin. Pregnancy history was not contributory. Neonatal anthropometric measurements were normal (birth weight = 3660 g, birth length = 52 cm, head circumference = 36 cm). At birth male-appearing external genitalia were observed but no structures were palpable in the scrotum (see Fig. 1). At age 3 weeks, an abdominal ultrasound was performed at the local hospital, showing the presence of an immature uterus and the absence of gonads. On that occasion the parents reported the infant’s repeated vomiting at home, and weight loss was noted on physical examination. Blood tests showed hyponatremia (Na 117 mEq/L; normal range, 136-146 mEq/L), hyperkalemia (K 7.1 mEq/L; normal range, 3.5-5 mEq/L), high ACTH (78.14 pg/mL; normal range, 7.2-63.3 pg/mL), and low cortisol levels (3.30 mcg/dL; normal values > 5 mcg/dL). A clinical diagnosis of CAH was suspected, and the onset with severe hyponatremic dehydration, hyperkalemia with failure to thrive in the first weeks after birth was suggestive of salt-wasting crisis.

The neonate was promptly transferred to our hospital where intravenous (iv) rehydration with sodium chloride and iv hydrocortisone was started, followed by an improvement in general condition and electrolyte normalization. Clinical evaluation showed a penile clitoris, urethral meatus at tip of the phallus, and scrotum-like labia (Prader 5). Further testing revealed increased levels of 17-hydroxyprogesterone (17OHP) (>44 ng/mL; normal range, 0.8-5 ng/mL), androstenedione (10 ng/mL; normal range, 0.30-3.10 ng/mL), testosterone (7.44 ng/dL; normal value < 2.5 ng/mL), and renin (>550 μIU/mL; normal range, 4.2-59.7 μIU/mL) supporting the clinical suspicion of CAH in a girl. The chromosome study confirmed a normal 46XX karyotype. Specific genetic analysis revealed homozygous deletion of the *CYP21A2* gene, confirming the diagnosis of classic CAH caused by 21-hydroxylase deficiency (21OHD) in a female newborn. Permission to display the image was obtained from the parents.

### Case 2

A 17-year 3-month girl with nonclassic CAH was evaluated at age 7 years for premature pubarche, clitoral hypertrophy, and bone age advancement by 2 years. Menarche appeared at age 11 years and since then the patient reported menstrual irregularities.

A standard ACTH test was performed at age 7 years and 2 months showing insufficient peak cortisol values (9.7 mcg/dL; normal range > 18-20 mcg/dL) and high 17OHP values (26 ng/mL; normal range < 4 ng/mL). Biochemical diagnosis was genetically confirmed by the detection of the pathogenic variants c.-333C>T and c.290-13A>G in compound heterozygosity in *CYP21A2*. Conventional therapy with immediate-release hydrocortisone was started after diagnosis (at age 7 years and 2 months). Adherence to treatment was optimal.

At the last follow-up visit at age 17 years, the patient reported persisting oligomenorrhea. Adult height was at the lower limits of normal for age and sex (−1.8 SD), while normal weight and BMI were observed. Elevated levels of testosterone (35 ng/dL) and 17OHP (7.1 ng/mL) were detected.

### Case 3

**Figure 2.**
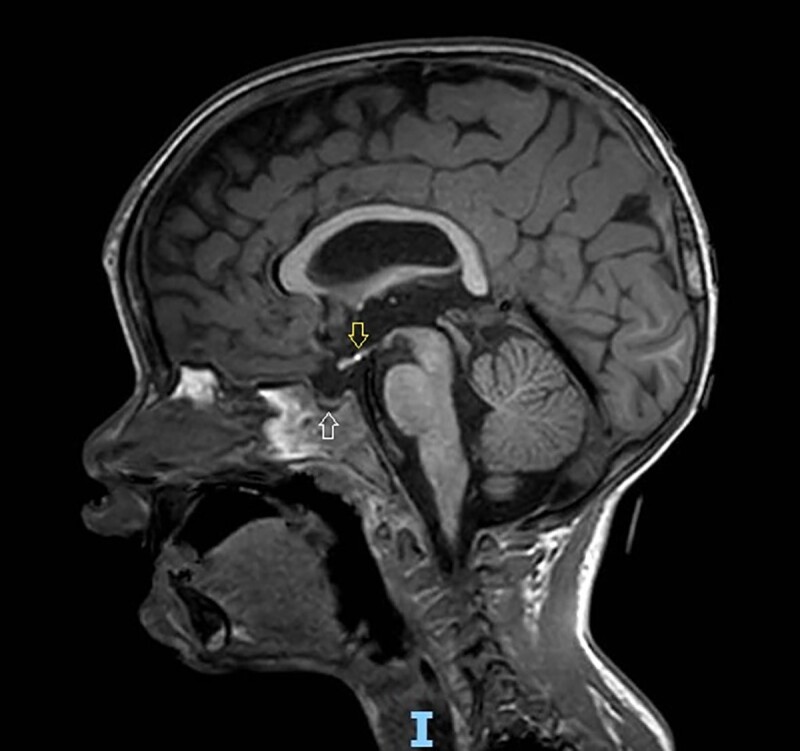
T1-weighted brain magnetic resonance imaging scan. Ectopic posterior pituitary at the level of median eminence (down arrow), thin pituitary stalk, and anterior pituitary hypoplasia (up arrow) in a 2-year boy with multiple pituitary hormone deficiency.

A 1-month-old boy was referred to our department for persistent hypoglycemia. The patient was born by emergency cesarean delivery at 38 weeks of GA due to non-reassuring electronic fetal monitoring. Intrauterine growth restriction had been reported starting in the first trimester of pregnancy. Neonatal birth weight was at the lower limits of normal for GA (birth weight = 2610 g), while other auxological data were normal (birth length = 48 cm; head circumference = 34 cm). Transfontanellar ultrasound revealed right ventricular dilatation and periventricular hyperechogenicity. Brain MRI showed an ectopic posterior pituitary, thin pituitary stalk, and anterior pituitary hypoplasia (Fig. 2).

Baseline ACTH and cortisol values were suggestive of central adrenal insufficiency (CAI) (baseline cortisol 0.41 mcg/dL, ACTH 40.1 pg/mL), and low-dose ACTH testing confirmed the diagnosis of CAI (cortisol peak value 5.2 mcg/dL); replacement therapy with galenic preparation of hydrocortisone was started at a dose of 10.4 mg/m^2^/day in 3 divided doses (1 mg + 1 mg + 0.5 mg). Impaired thyroid function compatible with the diagnosis of central hypothyroidism (free thyroxine 9.2 pg/mL [normal range, 9.3-17 pg/mL], thyrotropin 3.4 μU/mL [normal range, 0.2-4.2 μU/mL]) was detected (confirmed 2 times), and replacement therapy with levothyroxine was started after the administration of hydrocortisone. At age 1 year, growth hormone (rhGH) replacement therapy was also started because of severe growth retardation (length −3.5 SD, target height +0.2 SD, growth velocity in the last 6 months −3.9 SD) in the context of multiple pituitary hormone deficiency (MPHD) associated with congenital pituitary abnormalities identified by brain MRI.

## How Should These 3 Patients Be Managed?

### Background: Conventional Therapeutic Regimens

#### Glucocorticoid therapy

Patients with AI, regardless of etiology (primary or central), require long-term glucocorticoid replacement therapy (3-6). Maintenance dosing of glucocorticoids for replacement therapy is based on the secretory rate of cortisol under the control of an intact HPA axis. Data in children and adolescents indicate that the physiological secretion of cortisol in basal conditions may be as low as 5 to 6 mg/m^2^/day, without substantial changes during puberty (3, 6). Since the bioavailability of cortisol is reduced by gastric acids and first passage through the liver, the usual maintenance dose for glucocorticoid replacement should be adjusted above the estimated secretion rate (3, 6). Therefore, the dose 9 to 12 mg/m^2^/day of oral hydrocortisone represents a rational initial starting dose for individuals with primary adrenal insufficiency (PAI). Patients with CAI, which is often “partial,” usually require a lower dose. In both forms of AI, hydrocortisone is commonly administered every 8 hours (3 times daily), preferably with a higher dose in the morning to mimic physiological secretion (3, 5, 6). Unlike other forms of PAI, patients with classic CAH often require supraphysiological doses of glucocorticoid therapy to restore negative feedback on pituitary ACTH drive and to achieve adequate androgen suppression.

#### Mineralocorticoid therapy

In patients with PAI, mineralocorticoid replacement is recommended to correct aldosterone deficiency. Currently the only synthetic mineralocorticoid available is fludrocortisone, which is usually administered in doses ranging between 50 and 200 µg once or twice daily. Dose titration is based on clinical status (signs/symptoms suggestive of salt wasting, blood pressure, growth failure in infants), serum electrolytes, and plasma renin activity (7, 8).

The higher mineralocorticoid doses required by newborns and infants compared to older children may be explained by the physiological resistance to aldosterone in the kidney during this period (7, 9). Moreover, because of a lower glomerular filtration rate, a variable capacity of immature renal tubules to reabsorb sodium, and low sodium concentration in breast milk and formula, an additional supplemental dose of sodium chloride is required to maintain sodium balance in newborns and in early childhood.

The sodium chloride supplement requirement in newborns is 1 mmol/kg. However, in infants with salt-wasting CAH, this amount is inadequate and sodium supplementation is recommended, ideally using a saline solution alone or distributed in breast milk or artificial milk (5, 6, 9).

## Novel Treatment Approaches

In an effort to improve patient outcomes and minimize glucocorticoid exposure, new therapies targeting different levels of the HPA axis have been developed.

### Optimized Hydrocortisone Dosing in Children

Until 2018, the treatment for AI in newborns and infants was suboptimal because adult dosage formulations were used. In addition, these formulations were not appropriate to treat infants who require a daily dose of 10 to 15 mg/m² with single doses as low as 0.5 mg (4, 7, 8, 10-12). Crushed hydrocortisone tablets suspended in water were often used, although accurate dosing was not possible because hydrocortisone does not dissolve well in water and could adhere to plastic material when applied with syringes. Furthermore, to reduce the bitterness of hydrocortisone, a common practice was to mix the drug with sucrose (7). According to Gudeman et al (13), up to 25% of compounded batches did not meet the European Pharmacopeia acceptance criteria in terms of net drug mass/dosage or were incorrectly labeled.

In 2018, an immediate-release granule formulation of hydrocortisone, Infacort granules (ALKINDI SPRINKLE) was approved for the treatment of CAI and PAI in newborns and children. Infacort granules were developed to address the specific needs of pediatric patients, which include an appropriate dosing route, improved palatability, ease of administration for caregivers, dosing flexibility, and stability (Table 1) (22-24).

**Table 1.** New pharmacological therapies in adrenal insufficiency

	Drug	Approval	Data for pediatric ages	Route of administration	Strengths	Limitations
New formulations of glucocorticoids	Immediate-release hydrocortisone granules (Alkindi)	Approved for AI treatment from birth to age 18 y	Yes	Oral	—Oral administration —Appropriate dosing for infants and young children —Granule diameter <2 mm makes Alkindi safe from first days of life —Palatability	—Multiple doses needed —Does not mimic overnight cortisol levels —Granules must be swallowed immediately after dissolving them (in food or child's mouth) since bitter taste appears as soon as coating dissolves—usually a few minutes
	Dual-release hydrocortisone (Plenadren)	Approved for AI treatment in adults Not approved in children or adolescents aged <18 y	Yes (case series) (14)	Oral	—Oral administration —Once-daily dosing	—No control of overnight ACTH-driven androgen excess —Cannot be used for stress/sick-day dosing
	Modified-release hydrocortisone (Chronocort, Efmody)	Approved for CAH treatment in adults and in adolescents from age 12 y	No	Oral	—Oral administration —Fewer daily administrations than conventional hydrocortisone therapy —Mimics physiologic cortisol profile —Good control of androgen excess, good effect on fertility	—In stressful situations it is necessary to administer immediate-release hydrocortisone
	Continuous subcutaneous hydrocortisone infusion (pump)	Not approved for AI treatment	Yes (15-17)	Subcutaneous	—Mimics physiologic cortisol secretion —Good control of androgen excess	—Invasive —Risk of device malfunctioning —Costs
Aromatase inhibitors	Testolactone, letrozole	Not approved for AI treatment	Yes (18, 19)	Oral	—Oral administration —As adjunctive therapy can reduce glucocorticoid daily requirement —In coadministration with antiandrogen can prevent premature epiphyseal maturation with good effect on final height in children with CAH	—Risk of liver toxicity —Potential teratogenicity (not recommended in women of reproductive age and during pregnancy)
Antiandrogens	Flutamide	Not approved for AI treatment	Yes (18)	Oral	—Oral administration —As adjunctive therapy can reduce glucocorticoid daily requirement —In coadministration with aromatase inhibitors can prevent premature epiphyseal maturation with good effect on final height in children with CAH	—Need for associated glucocorticoid/aromatase inhibitor treatment —Risk of liver toxicity —Contraception recommended in women
Direct inhibitors of adrenocortical steroidogenesis	Nevanimibe	Not approved for AI treatment	No	Oral	—Oral administration —As adjunctive therapy can reduce glucocorticoid daily requirement	—Gastrointestinal side effects
	Abiraterone	Not approved for AI treatment	Ongoing (20)	Oral	—Oral administration —As adjunctive therapy, can reduce bone age advancement and allow normal growth	—Need for associated glucocorticoid treatment —Inhibition of sex hormones synthesis
HPA axis suppressors	CRF receptor antagonist (Crinecerfont)	Not approved for AI treatment	Yes (adolescents) (21)	Oral	—Oral administration —As adjunctive therapy may reduce glucocorticoid daily requirement	—Lack of long-term efficacy and safety data

Abbreviations: ACTH, adrenocorticotropin; AI, adrenal insufficiency; CAH, congenital adrenal hyperplasia; CRF, corticotropin-releasing factor; HPA, hypothalamic-pituitary-adrenal.

A phase 1 study of Infacort demonstrated good tolerability and palatability and bioequivalence to licensed hydrocortisone tablets in healthy volunteers (22). In 2018, Neumann et al (23) evaluated the absorption, palatability, and safety of Infacort in neonates, infants, and children younger than 6 years with CAI and CAH. This open-label, single-dose study evaluated 3 consecutive child cohorts (n = 24) with AI; cohort 1, children aged 2 to younger than 6 years (n = 12); cohort 2, infants aged 28 days to younger than 2 years (n = 6); and cohort 3, neonates aged 1 day to younger than 28 days (n = 6). The novel granule formulation was well tolerated, easy to administer to neonates, infants, and children, and showed good absorption, with cortisol levels at 60 minutes after administration similar to physiological cortisol levels in healthy children. Importantly, no safety concerns were reported in this study, and Infacort was easy to dose and palatable according both to participants and parents/caregivers (23).

In a prospective study of glucocorticoid treatment in 17 children with CAH and 1 with hypopituitarism aged from birth to 6 years and followed prospectively for 2.5 years, Neumann et al (24) demonstrated that the use of hydrocortisone granules resulted in an effective treatment of the children with AI, as demonstrated by the absence of adrenal crisis and a normal growth profile. No safety issues were detected. Over the course of the study, required daily doses of hydrocortisone remained stable and within the recommended range for CAH, with a reduction to the lower limit of the range at the end of the 2.5 years of observation.

### Modified-Release Hydrocortisone Formulations

New ways of replacing glucocorticoids have been developed in recent years. Two modified-release hydrocortisone formulations have been prepared: a dual-release formulation (Plenadren) and a modified-release formulation (Chronocort) (see Table 1) (14).

Plenadren consists of a dual-release tablet with an outer immediate-release hydrocortisone coating and an inner sustained-release hydrocortisone core. This therapy approximates the physiological cortisol profile in the early part of the day and received European Medicines Agency approval in 2011 for the treatment of AI in adults (25, 26). However, the overnight cortisol-free interval is not able to control the nocturnal ACTH-driven elevation of androgens that is characteristic of CAH, and data in these patients are lacking (7). A clinical trial assessing its potential use in adult patients with 21OHD CAH is ongoing (27).

In a recent study of 21 adults with CAI, Gasco et al (28) showed a significant reduction in waist circumference and BMI after 3 and 6 months of treatment with a dual-release formulation with an improvement of total quality of life (QoL) score. Younger patients, those with inadequately replaced GH deficiency at baseline, and those with lower QoL appeared to benefit the most from treatment. However, a worsening of high-density lipoprotein (HDL) cholesterol levels was observed during treatment. Larger, long-term studies are needed to confirm these preliminary findings.

Chronocort (Efmody) is an encapsulated formulation of hydrocortisone with a delayed-release coating that allows for delayed and sustained absorption. It is licensed in Europe and Great Britain for the treatment of 21OHD CAH patients aged 12 years or older. It has been specifically designed to mimic the circadian rhythm of cortisol, when administered twice daily (before onset of nocturnal sleep and on arising in the morning) to control androgen excess and chronic fatigue (7, 10, 29, 30). It is a multiparticulate formulation presented as a capsule and is available in doses of 5 and 10 mg (7, 10, 29, 30).

In a 6-month, nonrandomized, open-label phase 2 study, a twice-daily regimen of Chronocort in 16 adults with classic CAH demonstrated daytime adrenal androgen control, without serious side effects (31). Indeed, Jones et al (32), using 24-hour urinary steroid metabolome profiling, identified the differential effect of Chronocort and conventional glucocorticoid therapies on steroid excretion in adults with CAH. In particular, Chronocort reduced 17OHP and alternative pathway metabolites excretion to near-normal levels more consistently than other glucocorticoid preparations.

In a randomized phase 3 study, Merke et al (33) evaluated Chronocort vs standard glucocorticoids in 122 adult CAH patients. Although Chronocort was not more effective than standard glucocorticoid therapy based on the 24-hour serum 17OHP profile, patients treated with Chronocort showed improved biochemical control with decreased daytime hormonal fluctuations at 24 weeks and a reduction in median daily steroid dose over time at the 18-month extension. Restoration of menses occurred in 8 patients; 3 women and the partners of 2 male patients became pregnant and gave birth successfully.

In a recent study, Tschaidse et al demonstrated that 6 months of Chronocort therapy decreased plasma renin activity, increased sodium levels, and significantly decreased 17OHP (34). Since 17OHP is a known mineralocorticoid receptor antagonist, the observed greater agonist action of the mineralocorticoid substitution may be due to lower levels of 17OHP (34).

The administration of Chronocort must be carried out twice a day. It is recommended to take a dose between a quarter and a third of the prescribed dose in the morning, and the remaining (between two-thirds and three-quarters of the total dose) in the evening.

It should be noted that slow-release formulations are not ideal for use as a stressing dose and patients “under stress conditions” may need an additional replacement of immediate-release hydrocortisone tablets.

### Continuous Subcutaneous Hydrocortisone Infusion

The HPA axis releases hormones in complex and dynamic ultradian pulses in the classic circadian rhythm. Although standard glucocorticoid replacement quantitatively replenishes adrenal hormones, it does not restore the normal cortisol biorhythm. This discovery led to the search for a more physiological cortisol replacement regimen with continuous subcutaneous hydrocortisone infusion (CSHI) (15-18, 35-40). In a single-center, open-label, phase 1/2 study comparing oral glucocorticoids with CSHI, Turcu et al (16) studied 8 adults with poorly controlled CAH. The authors found that, as a consequence of the marked morning rise in ACTH that occurs in patients treated with oral glucocorticoids, the increased levels of adrenal steroids, particularly of 11-oxygenated androgens, decline rather slowly throughout the day. In contrast, CSHI inhibits the early rise in ACTH, thus allowing ACTH-driven adrenal steroids to return closer to baseline levels before 1200 hours. Interestingly, testosterone was lowered by CSHI in women but increased in men, suggesting improved testicular function secondary to better disease control. Mallappa et al (17) evaluated the safety and efficacy of long-term CSHI in 5 adults with classic CAH and found reduced morning levels of ACTH, 17OHP, androstenedione, and progesterone, and increased whole-body lean mass and improved QoL. A reduction of testicular adrenal rest tissue and adrenal size, already observed in 1 of the 2 male patients at 6 months, remained stable at the end of the study. In one female patient, the size of an adrenal adenoma progressively decreased over the course of the study. Subjective improvement in hirsutism was reported by all 3 female patients.

Few case reports describe the efficacy of the hydrocortisone pump in adolescents with CAH and poor disease control (35-37). There are also sporadic case reports/case series on the efficacy of CSHI in individuals with CAI for whom conventional oral administration was contraindicated or unreliable and/or ineffective (38, 39). In particular, Cardini et al (39) demonstrated the effectiveness of CSHI in a 42-year-old woman with CAI and significant improvement in perceived QoL at a 14-month follow-up.

Larger long-term studies are needed to assess the benefit of the hydrocortisone pump compared to current treatment practice.

### New Ways to Suppress Adrenal Androgens

#### Aromatase inhibitors and antiandrogens

The combination of an aromatase inhibitor and an antiandrogen was proposed as an alternative approach to the treatment of CAH (see Table 1). High estrogens, aromatized from adrenal androgens, are the main cause of advanced skeletal maturation in children with CAH, leading to reduced adult height.

The concomitant administration of an aromatase inhibitor and an antiandrogen should prevent premature epiphyseal maturation by inhibiting androgen-to-estrogen conversion, avoiding the clinical effects of androgen excess through androgen receptor blockade (7, 18, 40). In addition to the routinely used fludrocortisone and glucocorticoids, this combination therapy represents a potential approach to achieve normal growth and development in 21OHD children with severely advanced bone age due to negative consequences of previous suboptimal treatment.

Merke et al (19) reported a 2-year-experience in 28 children with the classic form of 21OHD CAH treated with flutamide (an antiandrogen), testolactone (an aromatase inhibitor), and low hydrocortisone dose. In these patients, reduction of the glucocorticoid dose during flutamide and testolactone treatment caused an expected increase in androgen levels, while estrogens remained stable, presumably due to the effect of the aromatase inhibitor. Despite this, growth and skeletal maturation rates decreased significantly during treatment, demonstrating the effectiveness of these drugs in controlling the growth acceleration characteristic of CAH (19).

In a retrospective study including 15 patients with 21OHD, Xi et al (41) showed that the addition of letrozole (an aromatase inhibitor) to gonadotropin-releasing hormone and/or GH therapy for at least 1 year significantly delayed bone maturation and increased the predicted adult height. However, data on adult height and long-term follow- up data are still lacking. A trial on the efficacy of letrozole and flutamide, and reduced hydrocortisone dose or conventional treatment is ongoing in children with CAH (42).

#### Abiraterone acetate

Abiraterone acetate is a prodrug for abiraterone, a potent cytochrome P450 family 17 subfamily A member 1 (CYP17A1) inhibitor used to suppress androgen synthesis in the treatment of prostate cancer. Its use in the management of CAH allows for a reduction in glucocorticoid dose and maintenance of normal serum androgens despite diminished ACTH suppression (see Table 1) (7, 10). Auchus et al (20) showed that abiraterone acetate—added to hydrocortisone for 6 days—normalized androgen excess in women with classic 21OHD, with an excellent short-term safety profile.

A phase 1/2 multicenter study to assess the efficacy and safety of abiraterone acetate as adjunctive therapy is ongoing in prepubertal children with classic 21OHD CAH. Phase 2 will determine if, over 24 months, this treatment delays bone age advancement and thus improves adult height prognosis (43).

#### Nevanimibe

By impairing all adrenocortical steroidogenesis, this drug has the potential to be used as an add-on therapy allowing for physiologic glucocorticoid replacement by removing hyperandrogenism risk (see Table 1). In an early phase 2 study in 10 adults with uncontrolled classic CAH, 2-week interval treatments of nevanimibe resulted in a decrease of 17OHP levels (44).

### Hypothalamic-Pituitary-Adrenal Axis Suppression

#### Corticotropin-releasing factor receptor antagonists

By acting directly on the pituitary to decrease ACTH, corticotropin-releasing factor (CRF) receptor antagonists such as crinecerfont could effectively reduce adrenal steroid production, avoiding the need for supraphysiologic doses of glucocorticoids (45). Their use in addition to hydrocortisone may allow glucocorticoid reduction (see Table 1 and Fig. 2) (21). Promising results on the use of CRF antagonists are reported in adults (21) and in adolescents (46), with a decrease in 17OHP, ACTH, and androstenedione levels.

#### Adrenocorticotropin and adrenocorticotropin receptor antagonists

Monoclonal antibody-based targeted therapy has been investigated in preclinical studies of ALD1613, a novel, long-acting monoclonal antibody directed toward ACTH (7, 47, 48). Pharmacokinetic-pharmacodynamic studies in wild-type rats showed that ALD1613 significantly reduced plasma corticosterone levels in a dose-dependent manner (47).

A selective, nonpeptide melanocortin type 2 receptor (MC2R) antagonist demonstrated suppression of corticosterone and reversal of ACTH exposure in rats (5). Based on these encouraging preclinical data, a double-blind, randomized, placebo-controlled phase 1 study on CRN04894, an oral MC2R antagonist, was started (49). Preclinical development of other MC2R peptide antagonists is ongoing (50, 51).

### Bilateral Adrenalectomy

In a systematic review and meta-analysis, MacKay et al (52) identified 48 cases of bilateral adrenalectomy for CAH with patients aged from 4 months to 56 years at surgery. The most common indications for surgery were the inability to control hyperandrogenism, virilization, Cushing syndrome, and infertility in women. Most patients (74%) reported symptomatic improvement after adrenalectomy. This included improvements in features of hyperandrogenism, recommencement of menses or onset of menarche, increased growth velocity, weight loss, and positive fertility outcomes (52). Adrenalectomy is not commonly performed as a therapeutic option for CAH. Concerns include surgical risk, loss of protective adrenal function, and risk of recurrent virilization due to adrenal rest tissue (7, 52, 53).

### Chemical Adrenolytic Therapy

Mitotane has a cytotoxic effect on tumor cells but also antisecretory effects on adrenal cells (7, 54). Currently, it is the only approved adrenolytic drug in the treatment of postoperative or inoperable adrenocortical carcinoma (54, 55). Its use is also described, off label, in the management of Cushing syndrome (56) and in CAH adults with testicular adrenal rest tumors (TARTs) associated with infertility unresponsive to intensified glucocorticoid therapy (57, 58).

Mitotane efficacy in restoring fertility was first reported in a 29-year-old classic CAH patient with bilateral TARTs, hypogonadism, and azoospermia (57).

A retrospective study on long-term effects of mitotane treatment in 5 patients with classic CAH and TART-associated infertility demonstrated its ability to reduce tumor size and improve testicular function (58). Chemical adrenolytic therapy has not been studied in women with CAH (probably because of its potential teratogenic effects) nor in children.

In summary, mitotane can be considered as a last therapeutic option only in selected cases as in male adults with 21OHD and azoospermia associated with TARTs who are unresponsive to intensified glucocorticoid treatment (7).

### Cell-Based and Gene Therapies

Adrenocortical cell transplantation or gene therapies are attractive novel approaches for the management of PAI since they allow a restoration of homeostatic cortisol secretion (59).

Different cell types, such as adult stem cells, embryonic stem cells, or induced pluripotent stem cells, have been successfully reprogrammed to generate cells with steroidogenic potential in vitro (60, 61). However, this approach has been little investigated in vivo. Experience with adrenal cell transplantation for PAI is, at present, predominantly preclinical, and mainly centered on murine studies. The adrenal cortex is a high-turnover organ, and thus failure to incorporate progenitor cells within a transplant will ultimately result in graft exhaustion (59, 62). Current technologies make cell therapy achievable, but they need some improvements such as cell isolation for allogeneic transplant, cell production from adult cells—using reprogramming or lineage conversion to allow a suitable adrenal-like steroidogenesis profile or use of devices to provide an appropriate environment for cell growth (59).

Gene therapy aims to correct the defect by replacing or repairing genes carrying pathogenic variants (59). Preclinical studies have reported promising results relying on adeno-associated virus as the leading vehicle for gene therapy (63-70). However, these preclinical studies have not shown a long-term restoration of normal steroidogenesis (63-70). The reason has to be found mainly in the continuous renewal of adrenal gland cells by stem cells that are located at the adrenal capsule (70). A phase 1/2 human clinical trial of the Safety and Efficacy of Gene Therapy in adults with 21OHD is ongoing (71).

## Back to Our Patients

### Case 1

Our patient was discharged after 2 weeks of hospitalization on therapy with oral hydrocortisone (20 mg/m^2^/day in 3 divided doses), fludrocortisone (0.05 mcg/day), and oral supplementation with sodium chloride. At longitudinal follow-up, good general conditions and regular growth rate and development were observed. At age 11 months, corrective surgery of the urogenital sinus (total urogenital mobilization vaginoplasty, urethroplasty) and clitoral reduction were performed. At age 1 year the hydrocortisone galenic solution was switched to Alkindi, and she is currently on replacement therapy with Alkindi (11 mg/m^2^/day in 3 divided doses) and fludrocortisone (0.05 mcg/day). During the 4-year-follow-up, the girl has been in good health, and the parents report excellent adherence to the therapy (usually administered with soft food). The girl has never had episodes of adrenal crisis as her parents were well educated on the need to increase the dose of hydrocortisone in case of stressful conditions. At the last follow-up visit at age 4 years, the girl appeared in good clinical status, showing normal growth parameters (height = 95.2 cm, −1 SD) for age and sex (Target height is −0.3 SD); weight 14.9 kg, 24th percentile for age and sex; BMI 16.4, (0.44 SD), and good hormonal control (17OHP 0.10 ng/mL, testosterone <2.5 ng/dL).

### Learning Points

- CAH should be suspected in all newborns with ambiguous genitalia. Early diagnosis prevents mortality and morbidity because of the life-threatening consequences of adrenal crisis.
- Children with CAH require regular, careful monitoring of signs/symptoms and growth to optimize replacement therapy.
- Patient education is essential and aims for the patients and their parents to be able to identify circumstances of increased risk and the first signs of adrenal crisis, to know when and how to increase treatment with oral glucocorticoids in case of stress, and to carry the necessary medications for their treatment in case of emergency.
- All patients must be equipped with a medical alert bracelet and emergency medical card to ensure that health-care providers are aware of their condition and can administer hydrocortisone via intramuscular or iv injection in case of emergency.

### Case 2

To improve disease control, the patient was switched to modified-release hydrocortisone therapy with Efmody at the same previous daily dose (14 mg/m^2^/day) divided in 2 doses: three-quarters of the daily dose was taken in the evening at bedtime, and the rest in the morning. Restoration of menstrual cycles was reported after 4 months of follow up; the patient also reported a feeling of well-being and improved QoL.

### Learning Points

- Efmody requires fewer daily administrations than conventional hydrocortisone therapy, an advantage that is crucial for improving adherence in adolescents and young adults.
- Efmody has been reported to have a good effect on menses restoration—as in our patient—and potential fertility following the reduction of androgens.
- In stressful situations, it is necessary to administer an immediate release hydrocortisone dose, either orally or by parenteral route, based on stress severity.
- A timely transition to adult services is essential to ensure good disease control and to avoid the long-term consequences of glucocorticoid use on bone, metabolism, and cardiovascular risk.

### Case 3

As soon as the baby completed weaning and was able to eat soft foods, the hydrocortisone galenic solution was replaced with Alkindi to improve palatability and ensure ease of administration for caregivers. The granules were administered to the patient with a spoon mixed with yogurt—making sure they were administered immediately after mixing—or sprinkled directly into his mouth. The daily hydrocortisone regimen (total daily dose of 10.9 mg/m^2^/day) was achieved easily. The parents reported good adherence to therapy and good hormonal control was observed at follow-up visits. At the last follow-up evaluation at age 4 years the patient was on hydrocortisone therapy at a dose of 8.3 mg/m^2^/day: Auxological measures were normal (height −0.7 SD; weight 20th percentile), and blood pressure and electrolytes were normal.

### Learning Points

- Ectopic posterior pituitary and stalk abnormality predict severe GH deficiency and MPHD. Such MRI findings should alert the clinician to the risk of evolving pituitary deficits over time and the need for careful follow-up in patients with suspected clinical signs of AI.
- In individuals at risk of MPHD, thyroid hormone replacement therapy must be started only after the treatment with hydrocortisone, as it may trigger adrenal crises in patients with untreated AI.

## Discussion

The 3 cases presented here raise important considerations on the management of children and adolescents with AI and on the effect of treatment on outcomes, adherence, and QoL. Patients with chronic AI receiving standard replacement therapy show a significantly reduced subjective QoL, regardless of age, sex, and primary or central etiology (3, 10, 72-77).

In a study including adults with CAI and PAI, Hahner et al (74) showed that patients with CAI exhibited significantly higher impairment in subjective health status on some subscales, suggesting that MPHD, which is present in almost all CAI patients, could cause subjective health status to worsen.

In a study including 92 adults with MPHD, Kao et al (78) showed that patients with MPHD had less education, more unemployment, lower marriage rates and incomes, and fewer children than healthy controls (*P* < .05 for each). MPHD patients scored significantly lower than controls in all QoL domains (*P* ≤ .001) and psychosexual function (*P* < .001) (78).

It should be considered that the underlying diagnosis (congenital hypopituitarism, brain tumors) and the need to take different drugs throughout the day have a key role in the QoL of MPDH patients (78). A recent large study in 104 children with CAH aged 12.7 ± 3 SD in 14 UK centers showed that children with CAH had worse QoL than healthy controls. The authors showed a correlation between higher weight SD/lower height SD with lower QoL and lower QoL in girls with evidence of overtreatment (79). In a recent study including patients with CAH in the Middle East (mean age 11.5 ± 4 years in boys, mean age 11.7 ± 2 years in girls), Shafaay et al (80) showed that patients with CAH had reduced QoL in all domains, particularly in the pain/discomfort and anxiety/depression domains, as well as lower total health scores compared to healthy controls. The effect of AI on health status is reflected in a high incidence of restrictions in daily life associated with the diagnosis of AI (7, 10, 74). The difficulty of precisely titrating conventional glucocorticoid preparations with the aim of balancing on one hand the risk of undertreatment—which can lead to the development of adrenal crisis and androgen excess—and on the other hand the risk of excessive treatment, seems to be one of the main causes of the deterioration of health status in individuals with CAH (7, 10). Although the acute sequelae, in particular life-threatening adrenal crisis, are the most feared by the parents, patients and parents should be warned of the long-term metabolic consequences that overtreatment can cause (81, 82). Long-term studies on growth in children with the most common form of CAH, 21OHD CAH, have shown that average adult height is reduced, presumably because of a combination of androgen and glucocorticoid excess (83-85). Torky et al (86) assessed the risk of cardiovascular and metabolic morbidity in a longitudinal study of CAH patients followed for a minimum of 5 years during both childhood and adulthood (n = 57) at the National Institutes of Health. Compared to the US population, patients with CAH had a higher (*P* < .001) prevalence of obesity, hypertension, insulin resistance, fasting hyperglycemia, and low HDL during childhood and obesity (*P* = .024), hypertension (*P* < .001), and insulin resistance (*P* < .001) during adulthood. Increasing the dose of mineralocorticoids was associated with hypertension (*P* = .0015) and low HDL levels (*P* = .0021) in children (86). Furthermore, the consequences of the disease in adulthood include gonadal dysfunction related to anovulation in women and to the development of adrenal rest tissue in men and psychiatric disorders including anxiety and depression, which may be related to the discomfort of living with a chronic illness, with the fear of adrenal crises and the side effects of treatment (87). Attempts to tailor hydrocortisone dose and to replace cortisol secretion in the most physiological way have recently shown substantial progress. Alkindi represents a therapeutic breakthrough for infants with congenital hypopituitarism and CAH who require tailored hydrocortisone therapy from the first days of life. As infants and children with CAI require lower doses of hydrocortisone than those with CAH, the need to adjust the dose is particularly important for this group of patients. The starting dose of hydrocortisone is 9 to 12 mg/m^2^/day divided into 3 to 4 doses and modified with age. It is mandatory to educate and inform families that in case of illness or stress (sick day rules), doses of hydrocortisone should be doubled or even tripled. In case of emergency or vomiting with suspected adrenal crisis, intramuscular hydrocortisone should be administered. The oral dose depends on age (<1 year 25 mg, 1-5 years 25-50 mg, >5 years 100 mg) and in case of hospitalization iv hydrocortisone at the dose of 1 to 2 mg/kg every 4 to 6 hours should be administered; oral glucose should also be administered to correct any associated hypoglycemia (88). The modified-release formulation, Chronocort, approved in adolescents with CAH aged 12 years or older, showing the benefits of fewer administrations and better hormonal control than conventional therapies, has a significant effect in clinical practice. Although there are limited data available on modified-release hydrocortisone formulations in individuals with CAI, these formulations could simplify daily life for these patients by requiring fewer administrations. The development of personalized cellular and gene therapies, able to reactivate physiological cortisol secretion, represents the next direction.

## Data Availability

Original data generated and analyzed during this study are included in this published article or in the data repositories listed in “References.”

## Abbreviations

17OHP: 17-hydroxyprogesterone
21OHD: 21-hydroxylase deficiency
ACTH: adrenocorticotropin
AI: adrenal insufficiency
BMI: body mass index
CAH: congenital adrenal hyperplasia
CAI: central adrenal insufficiency
CRF: corticotropin-releasing factor
CSHI: continuous subcutaneous hydrocortisone infusion
GA: gestational age
GH: growth hormone
HDL: high-density lipoprotein
HPA: hypothalamic-pituitary-adrenal
iv: intravenous
MC2R: melanocortin type 2 receptor
MPHD: multiple pituitary hormone deficiency
MRI: magnetic resonance imaging
PAI: primary adrenal insufficiency
QoL: quality of life
TARTs: testicular adrenal rest tumors
